# Toe-In and Toe-Out Walking Patterns and Lateral Wedge Insoles: A Musculoskeletal Simulation and Probabilistic Modelling Assessment of Medial Tibiofemoral Cartilage Mechanics

**DOI:** 10.3390/life15111677

**Published:** 2025-10-28

**Authors:** Jonathan Sinclair, Guoying Zhang

**Affiliations:** 1 Research Centre for Applied Sport, Physical Activity and Performance, School of Health Social Work & Sport, University of Lancashire, Preston PR1 2HE, UK; 2 Haiyuan College, Kunming Medical University, Kunming 650500, China

**Keywords:** medial tibiofemoral compartment, musculoskeletal simulation, lateral insoles, gait modification, metabolic power, osteoarthritis

## Abstract

This study examined lateral wedge insoles and altered foot progression angles on medial tibiofemoral loading and long-term cartilage failure risk. Fifteen healthy male participants walked under four conditions: neutral gait, lateral wedge insoles, toe-in and toe-out gait. Three-dimensional kinematics were captured using an eight-camera system, and ground reaction forces were measured via a piezoelectric force plate. Musculoskeletal simulation analysis quantified tibiofemoral compressive forces, cartilage stresses, strains, and whole-body metabolic power. Probabilistic modelling was employed to estimate the probability of cartilage failure. Comparisons across the four gait conditions employed linear mixed-effects models with repeated measures. Peak compressive forces, stresses and strains were significantly larger in the neutral (force = 2.68 BW, stress = 2.61 MPa & strain = 0.22), compared to toe-in (force = 2.51 BW, stress 2.47 MPa & strain = 0.21) and toe-out (force = 2.43 BW, stress 2.40 MPa & strain = 0.21) conditions. Medial tibiofemoral cartilage failure probability was also significantly larger in the neutral condition (14.04%) compared to toe-in (10.66%) and toe-out (7.89%) conditions. Whole-body metabolic power was also significantly greater in the toe-out (9.74 W/kg) condition compared to neutral (9.32 W/kg) and lateral insoles (9.36 W/kg). The findings suggest that toe-in or toe-out walking may reduce medial tibiofemoral osteoarthritis risk. However, the greater metabolic demand of toe-out walking may limit its long-term feasibility and practicality as a preventive intervention.

## 1. Introduction

Knee osteoarthritis (OA) is the most prevalent form of lower-limb OA and a leading contributor to disability worldwide [1], imposing substantial economic burdens on healthcare systems [2]. The condition is progressive and degenerative and is characterised by a gradual loss of articular cartilage within the knee joint [3]. Those affected frequently experience persistent ongoing pain and stiffness [4].

Epidemiological data indicate that approximately 10% of people over 55 years of age have knee OA [5], with more than 90% of cases affecting the medial tibiofemoral compartment [6]. Although the mechanisms underlying disease onset and progression are complex, excessive mechanical stress on tibiofemoral cartilage during gait and daily movement is considered a central factor [7]. Susceptibility is further increased by risk factors such as ageing, genetic predisposition, obesity, and previous injury [7].

Given the substantial prevalence of medial tibiofemoral OA and its link to abnormal joint loading, several conservative strategies have been developed to reduce medial joint stresses. Because direct measurement of tibiofemoral contact forces is difficult, the external knee adduction moment is widely used as a surrogate for medial compartment loading [8,9]. A common intervention is the application of lateral wedge insoles [10], which aim to medially displace the centre of pressure (CoP) and shorten the knee adduction moment lever arm [11]. The literature, however, reports mixed outcomes: many studies have shown that lateral wedges reduce the knee adduction moment during walking [12,13,14,15,16,17,18,19] and stair ascent [20], whereas others have observed no reduction [21,22]. Similarly, while some clinical trials have reported improvements in pain and function [23,24,25], others found limited or no benefit [26,27,28,29].

In addition to insoles, gait modification has been explored as a means of attenuating the knee adduction moment [30]. The most common approach is altering the foot progression angle, by either externally (toe-out) or internally (toe-in) rotating the foot [30]. Both strategies seek to influence the mediolateral CoP location and thereby alter knee joint loading [31]. Previous investigations indicate that toe-out gait [31,32,33,34,35] and toe-in gait [36] can reduce the magnitude of the knee adduction moment. Direct comparisons between the two strategies remain scarce, with Lynn and Costigan [37] demonstrating reductions in knee adduction moment with toe-out walking but not toe-in. Conversely, Van den Noort et al. [38] and Khan et al. [30] reported that toe-in gait reduced loading in early stance, while toe-out was more effective during late stance. These studies also showed that toe-in walking reduced the adduction moment impulse, capturing both magnitude and duration of medial loading. Simic et al. [39] found reductions at both early and late stance with toe-in gait, but in their study, toe-out gait produced a significant decrease in adduction moment impulse. Beyond biomechanical analyses, intervention studies suggest that toe-out gait can reduce pain and improve function in individuals with medial tibiofemoral OA [31,40], though no long-term clinical evaluation has been performed for toe-in gait. Nevertheless, as these strategies alter habitual gait patterns, they may increase metabolic and neuromuscular demands [31]. Since humans are evolutionarily optimized to minimize energy expenditure during locomotion, even small increases in metabolic cost could impede daily adoption and limit long-term sustainability outside the laboratory.

It is important to note that the knee adduction moment, although widely used, provides only an indirect approximation of medial tibiofemoral joint loading [41]. While it has been associated with medial knee cartilage degeneration [8,9], it does not directly measure tibiofemoral contact forces [42]. Data from instrumented prostheses indicate only weak to moderate associations between the knee adduction moment and actual medial tibiofemoral contact forces [43]. Herzog et al. [42] demonstrated that muscle forces play the dominant role in determining joint loading. Recent advances in musculoskeletal simulation now allow muscle-driven estimates not only of joint contact forces but also of whole-body metabolic power [44], providing a more comprehensive assessment of gait interventions. To date, such simulation approaches have not yet been adopted to examine the effects of lateral insoles and foot progression modifications on the metabolic demands of walking and medial tibiofemoral joint contact loading.

Challenges also persist in quantifying how different gait strategies and joint loading conditions contribute to the onset and progression of OA [45]. Computational probabilistic modelling of cartilage mechanics has emerged as a promising method for estimating the lifetime risk of OA under repeated loading conditions [46]. The principal advantage of this approach is its ability to estimate long-term risk of OA, allowing the effects of prophylactic interventions to be evaluated in young and healthy individuals, prior to the onset of pathological changes. However, this approach has not yet been used to investigate the influence of lateral wedge insoles or foot progression angle alterations on medial tibiofemoral cartilage failure probability.

Accordingly, the present study aimed to examine whether toe-in gait, toe-out gait, and lateral wedge insoles reduce medial tibiofemoral cartilage loading mechanics and lifetime failure probability compared with natural gait. Furthermore, this investigation also aimed to examine the metabolic demands of walking in the aforementioned conditions. It was hypothesized firstly that all three interventions would decrease medial tibiofemoral loading and probability of failure compared to natural gait, with no significant differences between them and secondly that toe-in and toe-out gait would increase the metabolic demands of walking.

## 2. Materials and Methods

### 2.1. Participants

Fifteen healthy male volunteers (mean  ±  SD: age 31.73  ±  4.96 years, stature 172  ±  6.0 cm, body mass 69.31  ±  9.92 kg, BMI 23.45  ±  2.78 kg/m^2^) were recruited through advertisements distributed across the university campus and surrounding community areas. A priori power analysis, informed by data from previous work [47] that identified a mean  ±  SD difference of 0.28  ±  0.60 BW in peak compressive medial tibiofemoral force across conditions, indicated that a sample size of 15 would achieve *α*  =  0.05 and *β*  =  0.80. Participants were eligible if they were aged 18–45 years, reported no knee pain or history of OA, had no known allergy or sensitivity to adhesive tape required for retroreflective marker placement, and were free from any other musculoskeletal pathology at the time of testing. Written informed consent was obtained in line with the Declaration of Helsinki, and the protocol received approval from the institutional ethics committee (STEMH 1013).

### 2.2. Experimental Insoles

The intervention involved commercially available full-length insoles (Slimflex Simple, High Density, Full Length, Algeos UK, Liverpool, UK). These were manufactured from ethylene-vinyl acetate with a shore A hardness of 65 and incorporated an 11 mm heel thickness, inclusive of the wedge. Each insole was designed with a continuous 5° lateral wedge spanning the full length.

### 2.3. Foot Progression Angles

To examine the effect of altered foot progression, target angles of 15° toe-in and 15° toe-out during stance were prescribed [30]. To aid consistency, the force plate was initially marked with two reference lines: one corresponding to 15° toe-out and the other to 15° toe-in for the right foot. These markers were removed before data collection, after which participants were instructed to reproduce the designated angles independently. This was considered an ecologically valid approach, since individuals adopting gait modifications in real-world walking would not ordinarily receive visual guidance or feedback [30].

The actual foot progression angles achieved were derived from force plate data by tracking the CoP trajectory. Angles were computed as the line connecting the CoP at initial contact and toe-off relative to the anterior (forward) direction, with positive values representing toe-out and negative values toe-in [48].

### 2.4. Procedure

Participants walked along a 22 m laboratory, ensuring contact with an embedded piezoelectric force plate (Kistler Instruments Ltd., Winterthur, Switzerland) using their right, dominant foot. The force plate captured data at 1000 Hz. Walking was performed at each participant’s self-selected velocity to maximise ecological validity and better reflect the long-term loading patterns produced by the gait conditions. Enforcing a fixed speed across trials was avoided, as this would not align with everyday walking behaviour; moreover, the probabilistic cartilage failure model described later in this section accounts for the total number of loading cycles.

Each participant completed five valid trials (one gait cycle per trial) in four gait conditions: (1) natural gait with self-selected foot angle, (2) toe-out progression, (3) toe-in progression, and (4) lateral wedge insoles with self-selected foot angle. A trial was accepted when the right foot made complete contact with the force plate and gait remained unaffected by the experimental setup. All participants wore the same footwear (Asics Gel Patriot 6) and were given a five-minute familiarisation period for each of the three intervention conditions [47]. The shoes had a mean mass of 0.265 kg, with a 22 mm heel thickness and 10 mm heel-toe drop. Ground reaction forces (GRFs) and motion data were collected concurrently. Kinematics were captured at 250 Hz with an eight-camera motion system (Qualisys Medical AB, Gothenburg, Sweden), and dynamic calibration was conducted prior to each session.

**Figure 1:**
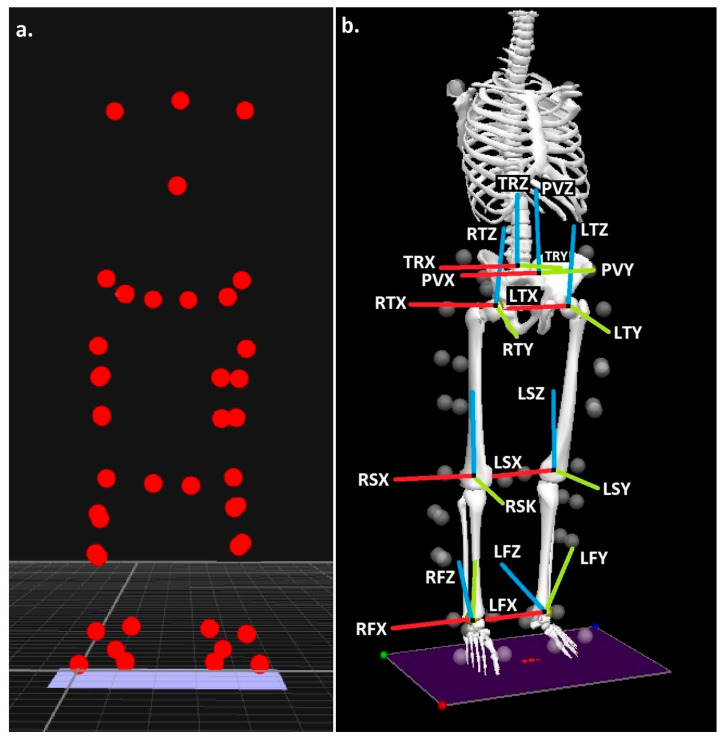
(**a**) Anatomical landmark locations and (**b**) modelled segments, with segment co-ordinate axes (R = right & L = left), (TR = trunk, P = pelvis, T= thigh, S = shank & F = foot), (X = sagittal, Y = coronal & Z = transverse planes).

Body segments were modelled with six degrees of freedom using the calibrated anatomical systems technique [49]. Anatomical reference frames for the thorax, pelvis, thighs, shanks, and feet were established through placement of retroreflective markers on C7, T12, and the xiphoid process, along with bilateral markers on the acromion processes, iliac crests, anterior and posterior superior iliac spines (ASIS, PSIS), medial and lateral femoral epicondyles, medial and lateral malleoli, greater trochanters, calcanei, and the first and fifth metatarsals (Figure 1a). Marker placement was carried out by a researcher with previously demonstrated excellent intra-rater reliability [50]. Knee and ankle joint centres were defined as the midpoints between the epicondyle and malleolar markers, respectively [51,52], while hip joint centres were estimated using a regression method based on ASIS marker positions [53]. Segment tracking was achieved with carbon-fibre clusters containing four non-collinear markers on the thighs and shanks, while the feet were tracked via markers placed on the calcaneus, first metatarsal, and fifth metatarsal. The pelvis was tracked using ASIS and PSIS markers, and the thorax using markers on C7, T12, and the xiphoid process. Static calibration trials were performed to establish anatomical marker positions relative to the tracking clusters. Segment coordinate systems were oriented following the right-hand rule: the *Z*-axis (transverse plane) aligned vertically from distal to proximal, the *Y*-axis (coronal plane) extended posterior to anterior, and the *X*-axis (sagittal plane) ran medial to lateral (Figure 1b).

### 2.5. Processing

The walking biomechanics dataset underwent four sequential stages of processing to estimate long-term medial tibiofemoral cartilage failure risk: (1) kinematic and temporal data, (2) simulation modelling, (3) medial tibiofemoral contact mechanics, and (4) probabilistic modelling of tibiofemoral cartilage.

#### 2.5.1. Kinematic and Temporal Data

Dynamic walking trials were first handled in Qualisys Track Manager (Qualisys Medical AB, Gothenburg, Sweden), where anatomical and tracking markers were identified and labelled. The resulting datasets were exported as C3D files into Visual 3D (C-Motion, Germantown, MD, USA) for subsequent analysis. The stance phase was defined duration over which the vertical GRF exceeded 20 N [54]. GRF signals were filtered with a 4th-order, zero-lag Butterworth filter with a 50 Hz cut-off, while marker trajectories were filtered at 6 Hz. These cut-off frequencies for kinetic and kinematic data were selected based on residual analysis [55]. Walking velocity was calculated within Visual 3D as the anterior linear velocity of the model’s centre of mass [56], and stride length was obtained as the change in anterior displacement of the foot’s centre of mass between consecutive ipsilateral footstrikes [56]. For each condition, the estimated number of daily loading cycles was computed by dividing the modelled daily walking distance by stride length.

The gait interventions (toe-in, toe-out, and lateral insoles) were designed to alter the mediolateral positioning of the CoP. Therefore, to evaluate their effect, the mediolateral location of the CoP relative to the foot’s centre of mass (mm) was calculated at the time of peak medial tibiofemoral contact force, determined by the subsequent analysis [57]. Positive values indicate that the CoP lies lateral to the foot’s centre of mass.

#### 2.5.2. Simulation Modelling

Kinematic and kinetic data from the stance phase were exported from Visual 3D into OpenSim v3.3 (Simtk.org). A validated musculoskeletal model was first scaled to each participant’s anthropometry. The model consisted of 12 body segments, 19 degrees of freedom, and 92 musculotendon actuators [58], enabling estimation of lower-limb muscle and joint loading. To minimise dynamic inconsistencies between measured GRFs and model kinematics, a residual reduction algorithm [44] was first applied. Muscle forces were then computed using the static optimization framework described by Steele et al. [59]. Since compressive tibiofemoral loads are primarily governed by muscle forces [42], the peak medial compressive tibiofemoral force (normalized to body weight, BW) was extracted via OpenSim’s joint reaction analysis, which used the outputs of static optimization as inputs [58]. Cumulative medial tibiofemoral loading was subsequently estimated by dividing the mean contact force by stride length, following the approach of Miller and Krupenevich [46].

Previous research has shown that, at the point of peak compressive tibiofemoral force, the vastus intermedius, vastus lateralis, and vastus medialis are the primary contributors to medial joint loading during walking [47]. To examine potential differences in peak medial tibiofemoral kinetics across gait conditions, muscle forces (BW) obtained through static optimization were extracted at the time of peak joint loading. These included the three aforementioned quadriceps muscles as well as other knee-crossing muscles: rectus femoris, biceps femoris long and short heads, semimembranosus, semitendinosus, and the medial and lateral gastrocnemius, which were subsequently entered into statistical analyses.

Metabolic cost minimization is a fundamental principle underlying the neural control of locomotion [60], with humans naturally favoring movement patterns that reduce metabolic energy expenditure [61]. Whole-body energy minimization is widely recognized as a key determinant of gait behavior, influencing both efficiency and sustainability [62]. Given that both toe-out and toe-in gait modifications have been shown to alter muscle force distributions in major lower extremity musculature [47], these strategies may lead to unsustainable walking patterns, potentially offsetting their intended benefits and reducing long-term adherence. Therefore, to fully understand the implications of these gait modifications, it was essential to also examine whole-body metabolic power using musculoskeletal simulation, providing a comprehensive assessment of their energetic efficiency and feasibility.

To accomplish this, the Computed Muscle Control (CMC) tool was applied within OpenSim [63,64]. The outputs from CMC were used as inputs to a modified version of the muscle energetics model described by Umberger et al. [65]. This implementation incorporated muscle-specific parameters, including proportions of slow- and fast-twitch fibers obtained from Johnson et al. [66], Garrett et al. [67], and Alway [68]. Muscle mass for each individual muscle was estimated from physiological cross-sectional area, optimal fiber length, and the fiber length associated with maximal isometric force capacity [69]. To maintain physiological validity, whole-muscle metabolic rate was constrained to remain non-negative, reflecting the principle that negative mechanical work does not contribute to ATP resynthesis [70]. Physiological cross-sectional area was calculated using a skeletal muscle density of 1059.7 kg/m^3^, consistent with mammalian muscle tissue [65], and maximal isometric force was estimated using muscle-specific tensions reported by Hamner and Delp [71] applied to measured cross-sectional areas [69].

Normalized whole-body metabolic power (W/kg) was obtained by adding a basal rate of 1.13 W/kg [72,73] to that generated from the Umberger et al. [65] probe in OpenSim. For statistical purposes, whole-body metabolic power was determined by integrating the estimated whole-body metabolic rate across the step duration using a trapezoidal approach [56].

In addition to whole-body values, muscle-level metabolic power (W/kg) was evaluated to examine the effects of the four gait modification conditions. Analysis was limited to the 21 muscles previously identified as the largest contributors to metabolic cost [74]: extensor digitorum longus, gluteus minimus, gluteus medius, gluteus maximus, psoas major, iliacus, rectus femoris, vastus intermedius, vastus lateralis, vastus medialis, adductor magnus, biceps femoris long and short heads, semitendinosus, semimembranosus, sartorius, adductor longus, tibialis anterior, medial and lateral gastrocnemius, and soleus. For muscles represented by multiple lines of action within OpenSim (gluteus minimus, gluteus medius, gluteus maximus, adductor magnus), outputs were combined for simplicity [58,74]. Muscle-level metabolic power was calculated by integrating each muscle’s metabolic rate across the step duration [56].

#### 2.5.3. Medial Tibiofemoral Contact Mechanics

Peak medial tibiofemoral compressive forces during the stance phase were applied to a medial knee contact mechanics model to estimate corresponding stress and strain within the tibiofemoral cartilage. This analysis was conducted in MATLAB (R2023b) using scripts adapted from the method of Miller and Krupenevich [46]. The modelling framework was based on the approach originally introduced by Nuño and Ahmed [75].

Within the model, the medial femoral condyle was represented by two convex arcs in the sagittal plane (anterior and posterior regions) and a single arc in the posterior coronal plane, while the tibial plateau was represented as a concave curve. The tibial arc radius in the frontal plane was fixed at 21 mm, and the sagittal radii of the femoral arcs were 35.0 mm (anterior) and 18.9 mm (posterior). The tibial component was held fixed, whereas the femoral component included two adjustable parameters: the vertical location of the knee flexion axis relative to the tibia and the knee flexion angle itself, consistent with Nuño and Ahmed [75]. Tibiofemoral cartilage was represented by an array of spring elements distributed across the tibial plateau, each with an unloaded thickness of 5.0 mm [76]. These elements were assumed to exhibit nonlinear elastic stress–strain properties [77]. Medial tibiofemoral contact stress (σ) and strain (ε) were then determined using Equations (1) and (2).

σ = −Mean tibiofemoral cartilage modulus * Log(1 − ε)(1)

ε = Cartilage element compression/modelled cartilage height(2)

Peak compressive stresses (MPa) and strains within the tibiofemoral joint were estimated using three key parameters: the elastic modulus of the loaded cartilage, the deformation of the contact elements under compression, and the total number of elements included in the model. The mesh comprised 7326 elements, corresponding to a 0.5 mm spacing and consistent with the defined tibial and femoral arc radii. The elastic modulus values assigned to the cartilage differed depending on meniscal coverage, with 8.6 MPa for femoral cartilage, 4.0 MPa for uncovered tibial cartilage, and 10.1 MPa for tibial cartilage beneath the meniscus [78]. The medial meniscus itself was given a modulus of 1.3 MPa and modelled as covering 46% of the tibial plateau surface [79,80]. A Poisson’s ratio of 0.45 was applied to both cartilage and meniscal tissues.

The knee flexion angle incorporated into the model was taken from the initial kinematic analysis at the instant of peak medial tibiofemoral contact force, derived from the joint force calculations. To establish loading, the vertical position of the knee flexion axis was first adjusted so that the cartilage elements were unloaded, and then gradually lowered until the predicted force matched the medial tibiofemoral contact force input. Because different radii were assigned to the anterior and posterior aspects of the medial femur, the proportion of cartilage engaged in loading varied as a function of flexion angle [81]. Translational mechanics were excluded from the model, in line with evidence that the medial femoral surface typically remains centered on the tibial plateau across knee flexion [82].

#### 2.5.4. Probabilistic Modelling of Tibiofemoral Cartilage

In line with the framework of Miller & Krupenevich [46], cartilage failure in the medial tibiofemoral compartment was defined as the onset of macroscopic plastic deformation, a hallmark of early osteoarthritic degeneration [83]. Since the median age of knee OA diagnosis is approximately 55 years, with 9.29% of the U.S. population affected symptomatically by that age [5], the modelling window extended across 37 years, representing the period from skeletal maturity at 18 years through to age 55 [84]. Failure probability was estimated using a probabilistic damage-repair approach [85,86,87], where compressive cartilage strain acted as the surrogate variable for cumulative tissue damage. Our prior analyses [88] have examined the sensitivity of peak compressive tibiofemoral strain, the principal predictor of cartilage failure in this model, by systematically varying each parameter within biologically and anthropometrically realistic ranges, while holding all others constant.

The probability of cartilage failure at the 37-year target point was determined as a cumulative outcome of cyclic loading, based on the modelled cartilage experiencing repeated stresses over a daily travel distance. Stride length values extracted from the kinematic and temporal data were incorporated into this calculation, as formalised in Equation (3).

Probability of cartilage failure = 1 − Exp − [(Volume of stressed cartilage/Reference stressed cartilage volume) (time/time until failure) ^Weibull exponent/Power law exponent^] (3)

In Equation (3), constant values were specified for the reference stressed cartilage volume (78.5 mm^3^), the Weibull exponent (14.3), and the power law exponent (12.9). The estimated time to cartilage failure was subsequently obtained from Equation (4).

Time to failure = (Power law coefficient * Stride length/Distance per day) (Weibull coefficient * ε) ^–Power law exponent^ (4)

In Equation (4), the predicted time to failure reflects the point at which 63.2% of simulated samples would be expected to fail under the imposed magnitude and frequency of cartilage strain. The model assumed a daily travel distance of 6.0 km, approximating the distance covered by completing 7000 steps per day, a value now widely regarded as beneficial for overall health [89]. For each gait condition, the daily number of loading cycles (steps) was calculated by dividing this distance by the stride length obtained from the kinematic and temporal data analysis. In the time-to-failure calculations, the Weibull coefficient (1.03), the power law coefficient (1.0), and the power law exponent (12.9) were applied as fixed constants. Parameter values for the Weibull and power law functions were adopted from Miller & Krupenevich [46], who fitted a power-law model for cycles to failure based on data from Riemenschneider et al. [90].

Equation (3) outlines the probability of cartilage failure in vitro. To account for the ability of living cartilage to partially repair strain-induced damage over time [91], the probability of recovery within the medial tibiofemoral cartilage was incorporated into the framework through Equation (5).

Probability of repair = 1 − Exp − [ − (time/time until repair) ^Cartilage repair exponent^] (5)

In Equation (5), the time to repair (fixed at 5.0 years) and the repair exponent (5.2) were specified based on values reported by Miller & Krupenevich [46]. The repair time was defined as the duration at which 63.2% of damaged cartilage cases would be expected to show recovery. Following the method outlined by Miller & Krupenevich [46], this repair mechanism was incorporated into Equation (3) by generating a probability density function that represents the instantaneous likelihood of failure across time. This approach is formalised in Equation (6).

Probability density function = (Volume of stressed cartilage * Weibull exponent/power law exponent * Reference stressed cartilage volume * time until failure) (time/time until failure) ^Weibull exponent/Power law exponent − 1^ exp [−(Volume of stressed cartilage/Reference stressed cartilage volume) (time/time until failure) ^Weibull exponent/Power law exponent^] (6)

To account for both failure and repair, the probability density function was multiplied by the cumulative likelihood that repair had not yet taken place (1 − probability of repair) and then integrated across time. This procedure is shown in Equation (7).

Probability of failure with repair = ∫ ^(time 0)^ [Probability density function * (1 − Probability of repair)] modelled distance between contact elements * time. (7)

Miller & Krupenevich [46] noted that this repair modelling approach may overestimate recovery in cartilage, since it assumes that damage occurring at any time point is fully restored after a duration equal to the sum of the damage and repair periods, thereby implying near-complete recovery of all damage.

### 2.6. Statistical Analyses

Mean values and standard deviations (SDs) were calculated for all biomechanical and cartilage failure outcomes. Differences between the four gait conditions were assessed using linear mixed-effects models with repeated measures [92]. The restricted maximum likelihood estimation was applied alongside compound symmetry, with gait condition specified as a fixed effect and participant-level random intercepts included [88]. Effect sizes (Cohen’s *d*) were reported and interpreted as small (*d* = 0.2), medium (*d* = 0.5), or large (*d* = 0.8) according to accepted guidelines [93]. Statistical analyses were performed in SPSS version 29 (IBM, SPSS, New York, NY, USA), with significance set at *p* ≤ 0.05.

## 3. Results

### 3.1. Kinematic and Temporal Data

Walking speed was greater in the neutral gait (*p* = 0.002, *d* = 0.97) and with lateral insoles (*p* = 0.007, *d* = 0.81) compared with toe-out. The CoP was shifted more laterally during toe-in relative to neutral (*p* = 0.001, *d* = 1.06), toe-out (*p* = 0.002, *d* = 0.95), and lateral insoles (*p* < 0.001, *d* = 1.58). Additionally, the neutral gait exhibited a more lateral CoP than both lateral insoles (*p* = 0.018, *d* = 0.69) and toe-out (*p* = 0.019, *d* = 0.69) (Table 1).

### 3.2. Simulation Modelling

Peak medial tibiofemoral compressive force was greater under the neutral condition compared with toe-in (*p* = 0.005, *d* = 0.85) and toe-out (*p* = 0.001, *d* = 1.09). Similarly, lateral insoles produced greater values than toe-in (*p* = 0.006, *d* = 0.84) and toe-out (*p* < 0.001, *d* = 1.64) (Table 2). Peak cumulative compressive force was also greater in the neutral condition relative to toe-in (*p* = 0.023, *d* = 0.66) and toe-out (*p* < 0.001, *d* = 1.78), while lateral insoles exceeded toe-out (*p* = 0.001, *d* = 1.11) (Table 2).

For muscle forces, vastus intermedius was greater in neutral compared to toe-in (*p* = 0.01, *d* = 0.76) and toe-out (*p* = 0.001, *d* = 1.06), in lateral insoles compared to toe-out (*p* < 0.001, *d* = 1.43), and in toe-in compared to toe-out (*p* = 0.038, *d* = 0.59). Vastus lateralis was greater in neutral compared to toe-in (*p* = 0.004, *d* = 0.89) and toe-out (*p* = 0.001, *d* = 1.03), and in lateral insoles compared to toe-out (*p* < 0.001, *d* = 1.21). Vastus medialis was greater in neutral compared to toe-out (*p* = 0.001, *d* = 1.05), in lateral insoles compared to toe-out (*p* < 0.001, *d* = 1.63), and in toe-in compared to toe-out (*p* = 0.001, *d* = 1.02). Rectus femoris was greater in toe-out compared to neutral (*p* = 0.041, *d* = 0.58), lateral insoles (*p* < 0.001, *d* = 1.36), and toe-in (*p* < 0.001, *d* = 1.62), with lateral insoles also exceeding toe-in (*p* = 0.018, *d* = 0.69) (Table 2).

**Figure 2:**
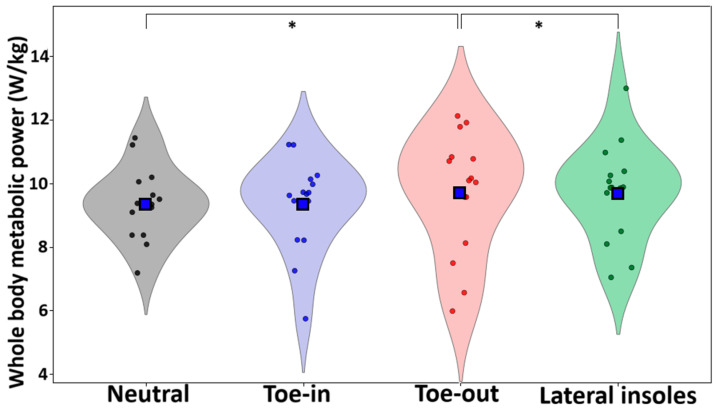
Violin plots for each condition. Blue squares represent the mean and * denotes significant differences at the *p* < 0.05 level.

Whole-body metabolic power was greater during toe-out walking compared to both neutral (*p* = 0.033, *d* = 0.61) and lateral insoles (*p* = 0.015, *d* = 0.72) (Figure 2). Muscle-level metabolic outputs are reported in the Supplementary Material (S1).

### 3.3. Medial Tibiofemoral Contact Mechanics

Peak medial tibiofemoral compressive stress was greater in the neutral gait compared with toe-in (*p* = 0.01, *d* = 0.76) and toe-out (*p* = 0.001, *d* = 1.08). Lateral insoles also produced greater stresses than toe-in (*p* = 0.005, *d* = 0.86) and toe-out (*p* < 0.001, *d* = 1.35) (Table 3). Similarly, peak medial tibiofemoral compressive strain was greater during neutral gait compared to toe-in (*p* = 0.01, d = 0.77) and toe-out (*p* = 0.001, *d* = 1.09), with lateral insoles again exceeding both toe-in (*p* = 0.005, d = 0.85) and toe-out (*p* < 0.001, *d* = 1.53) (Table 3).

### 3.4. Probabilistic Modelling of Tibiofemoral Cartilage

**Figure 3:**
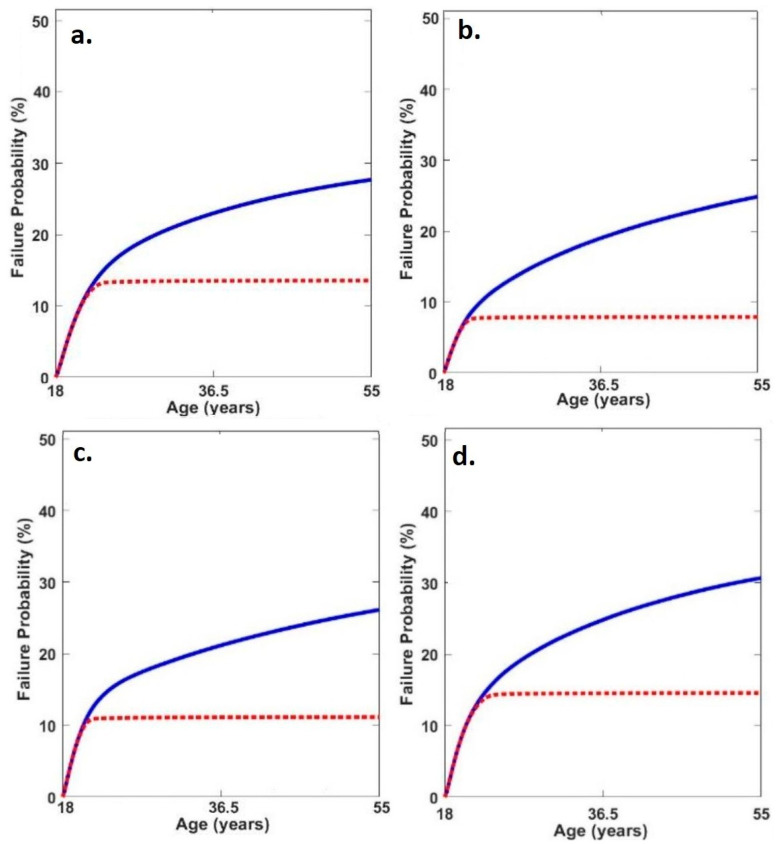
Average medial tibiofemoral cartilage failure time series probabilities in (**a**) neutral, (**b**) toe-out, (**c**) toe-in and (**d**) lateral insoles (red line = failure probability with adaptation and blue line = failure probability without adaptation).

When considering repair, the probability of medial tibiofemoral cartilage failure was greater in the neutral condition compared to both toe-in (*p* = 0.046, *d* = 0.56) and toe-out (*p* = 0.05, *d* = 0.55) (Table 4, Figure 3).

## 4. Discussion

Medial tibiofemoral OA is a common condition that imposes substantial economic burdens on healthcare systems and is frequently accompanied by persistent pain and stiffness. Our understanding of how altered gait mechanics and lateral insoles influence cartilage loading, long-term probability of OA and the energetic consequences of gait modifications remains poorly understood. The aim of this investigation was therefore to evaluate how toe-in gait, toe-out gait, and lateral wedge insoles affect medial tibiofemoral cartilage loading and the projected lifetime probability of cartilage failure. In addition, concurrent analysis of whole-body metabolic power was undertaken to provide insight into the potential long-term sustainability of these gait modifications. Together, these results are intended to provide new perspectives on the potential preventative and therapeutic roles of these strategies in supporting medial tibiofemoral cartilage integrity.

In line with the original hypothesis, both toe-in and toe-out gait patterns produced statistically significant reductions in medial tibiofemoral forces, stresses, and strains relative to a neutral walking pattern. By contrast, the lack of any significant reductions in cartilage loading with lateral wedge insoles was contrary to expectations. The findings with respect to toe-in and toe-out walking are consistent with prior work [31,32,33,34,35,36], which used the knee adduction moment as a principal outcome, whereas the findings for lateral insoles diverge from several earlier reports [12,13,14,15,16,17,18,19].

Although the precise mechanisms explaining these discrepancies remain uncertain, earlier research [47] identified vasti muscle forces as the strongest contributors to medial tibiofemoral joint loading during gait. This implies that the differences observed across the present experimental conditions may be attributable to alterations in vasti muscle activity. As vasti forces were significantly reduced during both toe-in and toe-out gait, it seems probable that the reductions in medial tibiofemoral loading arose from these kinetic adjustments. Despite all three interventions shifting the CoP location, only the toe-in and toe-out gait modifications demonstrated an ability to reduce the mechanical factors associated with medial knee OA risk.

Walking velocity was larger in the neutral condition, and given that medial tibiofemoral joint loading is affected by gait speed [94], some portion of the observed differences may reflect velocity-related influences. This also suggests that toe-out modifications may constrain preferred walking speed compared with unmodified gait. Moreover, cumulative medial tibiofemoral loading was significantly reduced under both toe-in and toe-out conditions, further supporting the initial hypothesis. Since stride length and daily loading cycles were not significantly altered, the observed reduction in cumulative load likely reflects reduced medial tibiofemoral loads per step.

Consistent in part with our original hypotheses, the present study found that the probability of cartilage failure was significantly decreased in the toe-in and toe-out gait conditions compared to the neutral gait. Given that the number of daily loading cycles did not differ between conditions, interpretation of the probabilistic failure framework [46] indicates that these reductions were likely driven by decreases in cartilage strain under the modified gait patterns. Altering the foot progression angle can improve pain and functional outcomes in individuals with established knee OA [31,40], and the current findings extend this evidence, suggesting that such gait modifications may also hold preventative potential, helping to reduce the risk of symptom onset. Considering the disabling pain, reduced mobility, and considerable healthcare costs associated with knee OA [1,2], toe-in and toe-out gait strategies may warrant inclusion as part of long-term musculoskeletal management aimed at preventing medial tibiofemoral OA.

In partial agreement with our hypothesis, the findings demonstrate that although both toe-in and toe-out gait modifications reduced medial tibiofemoral cartilage failure probability compared to the neutral condition, whole-body metabolic power was significantly greater in the toe-out condition. The muscle-level metabolic outcomes presented in S1 reveal distinct redistributions of muscular workload in response to the experimental gait modifications. Specifically, the toe-out gait condition was characterized by increased metabolic demands in the gluteal musculature, with a concurrent reduction in the energetic contribution of the vasti muscles. Conversely, the toe-in gait condition resulted in elevated metabolic activity within the adductor and the semimembranosus, coupled with a relative unloading of the gluteal muscles. As previous analyses have demonstrated that the hip adductors are the predominant contributors to the metabolic cost of walking [95], it is proposed that the elevated metabolic power observed in the toe-out gait condition may be attributable to increased metabolic demand placed on these muscles (i.e., gluteal muscles) during this condition. Given that metabolic cost minimization is a fundamental principle guiding the neural control of human locomotion [60], the metabolic penalty associated with toe-out gait may render this gait modification less sustainable over time, potentially diminishing the clinical utility of the toe-out gait as a preventative strategy for medial tibiofemoral OA.

The plausibility of the medial tibiofemoral mechanical outcomes observed in this work was evaluated by comparison with previous computational and in vivo investigations. Given the walking speeds tested, the peak medial tibiofemoral forces were consistent with those reported for participant K8L (2.59 BW at 1.39 m/s) from in vivo datasets [96]. Likewise, the cartilage strain values (0.23 at 1.52 m/s) and longitudinal failure probability for medial tibiofemoral cartilage in the neutral condition aligned closely with those reported by Miller & Krupenevich [46] (13.4%) and correspond with epidemiological incidence rates for medial tibiofemoral OA at age 55 [5].

Sex is a recognized independent risk factor for knee OA, with females exhibiting increased prevalence [97], and established sex-related differences in walking mechanics have also been reported [98]. Therefore, the generalizability of our findings to females remains uncertain; and future studies should therefore evaluate toe-in gait, toe-out gait, and lateral wedge insoles using probabilistic modelling in female cohorts. It is important to note that OA is recognised as a multifactorial disorder [99], with chronic, low-grade inflammation alongside mechanical loading, proposed as a key factor in its pathogenesis [100]. The computational framework applied in the present study did not incorporate potential influences from circulating adipokines, which could be considered a limitation. Future refinement of this modelling approach should aim to integrate both mechanical and biological determinants of OA into a pathophysiologically relevant probabilistic model, thereby enabling more comprehensive assessment of potential disease-modifying interventions. Moreover, directed by the present findings, future research should include longitudinal investigations to now provide the direct evidence necessary to substantiate the prophylactic potential of gait modification strategies. Finally, though familiarization was undertaken [47] alongside a repeated measures study design; a potential drawback is the novel exposure to different walking modalities. Neuromuscular demand of novel tasks can decline with repeated exposure, as motor recruitment becomes more efficient [101]; emphasizing the need for further longitudinal research.

## 5. Conclusions

Whilst previous studies have examined the biomechanical consequences of toe-in and toe-out gait adjustments and the use of lateral wedge insoles, their long-term influence has not previously been explored within an integrated musculoskeletal simulation and probabilistic modelling framework. The present work extends current knowledge by assessing how these gait strategies and lateral insoles influence the likelihood of medial tibiofemoral cartilage failure when compared with a neutral walking pattern. The analyses showed that cartilage forces, stresses, and strains were greater during neutral gait than under toe-in or toe-out conditions. Importantly, both toe-in and toe-out walking reduced the estimated probability of cartilage failure, although the toe-out pattern was accompanied by greater metabolic cost. These results indicate that altering foot progression to a toe-in or toe-out position may reduce medial tibiofemoral OA risk, though the additional metabolic burden of toe-out gait may limit its practicality as a long-term preventive strategy.

## Figures and Tables

**Table 1:** Initial kinematic processing values (mean and standard deviations).

	Neutral		Toe-In		Toe-Out		Lateral Insoles		Conditions
	Mean	*SD*	Mean	*SD*	Mean	*SD*	Mean	*SD*	
Foot progression angle (°)	3.51	5.86	−10.63	3.91	11.95	2.25	2.33	3.10	
Walking velocity (m/s)	1.54	0.11	1.49	0.15	1.49	0.11	1.56	0.12	**A, B**
Stride length (m)	1.71	0.10	1.72	0.11	1.74	0.12	1.71	0.09	
Daily loading cycles	7048.38	406.15	6997.73	413.27	6939.62	499.41	7055.44	382.94	
CoP position relative to foot centre of mass (mm)	1.39	5.12	8.45	8.05	−4.58	7.63	−1.03	4.25	**A, C, D, E, F**

Notes: A = Neutral significantly different from toe-out, B = Lateral insoles significantly different from toe-out, C = Lateral insoles significantly different from toe-in. D = Neutral significantly different from lateral insoles, E = Neutral significantly different from toe-in & F = Toe-in significantly different from toe-out.

**Table 2:** Musculoskeletal simulation values (mean and standard deviations).

	Neutral		Toe-In		Toe-Out		Lateral Insoles		Conditions
	Mean	*SD*	Mean	*SD*	Mean	*SD*	Mean	*SD*	
Peak compressive medial tibiofemoral force (BW)	2.68	0.47	2.51	0.46	2.43	0.46	2.69	0.49	**A, B, C, E**
Medial compressive medial tibiofemoral cumulative load (BW/m)	0.85	0.09	0.81	0.13	0.80	0.09	0.84	0.09	**A, B, E**
Vastus intermedius (BW)	0.51	0.09	0.41	0.15	0.35	0.17	0.46	0.19	**A, B, E, F**
Vastus lateralis (BW)	0.93	0.17	0.72	0.29	0.64	0.32	0.82	0.35	**A, B, E**
Vastus medialis (BW)	0.42	0.08	0.37	0.13	0.29	0.14	0.39	0.15	**A, B, F**
Rectus femoris (BW)	0.29	0.20	0.24	0.23	0.45	0.33	0.36	0.34	**A, B, C, F**
Biceps femoris long head (BW)	0.08	0.04	0.06	0.03	0.07	0.07	0.07	0.05	
Biceps femoris short head (BW)	0.02	0.02	0.02	0.01	0.03	0.04	0.01	0.01	
Semimembranosus (BW)	0.09	0.06	0.07	0.05	0.06	0.07	0.08	0.07	
Semitendinosus (BW)	0.02	0.01	0.02	0.01	0.01	0.02	0.02	0.01	
Lateral gastrocnemius (BW)	0.01	0.01	0.02	0.02	0.02	0.01	0.01	0.01	
Medial gastrocnemius (BW)	0.02	0.02	0.02	0.02	0.04	0.06	0.02	0.01	

Notes: A = Neutral significantly different from toe-out, B = Lateral insoles significantly different from toe-out, C = Lateral insoles significantly different from toe-in, E = Neutral significantly different from toe-in & F = Toe-in significantly different from toe-out.

**Table 3:** Medial tibiofemoral contact mechanics (mean and standard deviations).

	Neutral		Toe-In		Toe-Out		Lateral Insoles		Conditions
	Mean	*SD*	Mean	*SD*	Mean	*SD*	Mean	*SD*	
Peak compressive medial tibiofemoral stress (MPa)	2.61	0.64	2.47	0.66	2.40	0.64	2.64	0.68	**A, B, C, E**
Peak compressive medial tibiofemoral strain	0.22	0.05	0.21	0.05	0.21	0.05	0.22	0.05	**A, B, C, E**

Notes: A = Neutral significantly different from toe-out, B = Lateral insoles significantly different from toe-out, C = Lateral insoles significantly different from toe-in & E = Neutral significantly different from toe-in.

**Table 4:** Medial tibiofemoral cartilage failure probabilistic values (mean and standard deviations).

	Neutral		Toe-In		Toe-Out		Lateral Insoles		Conditions
	Mean	*SD*	Mean	*SD*	Mean	*SD*	Mean	*SD*	
Probability of failure with repair (%)	14.04	27.57	10.66	24.70	7.89	19.55	14.58	29.53	**A, E**

Notes: A = Neutral significantly different from toe-out & E = Neutral significantly different from toe-in.

## Data Availability

The raw data supporting the conclusions of this article will be made available by the authors on request.

## Supplementary Materials

The following supporting information can be downloaded at: https://www.mdpi.com/article/10.3390/life15111677/s1.
