# Supplementary material for: Toe-In and Toe-Out Walking Patterns and Lateral Wedge Insoles: A Musculoskeletal Simulation and Probabilistic Modelling Assessment of Medial Tibiofemoral Cartilage Mechanics
Source: Life (Basel). 2025 Oct 28;15(11):1677. doi: 10.3390/life15111677 (PMC12653958; doi:10.3390/life15111677)
Supplement: Supplementary file 1 [file life-15-01677-s001.zip › life-3932805-supplementary.pdf]

Supplementary Table S1. Muscle metabolic values (Mean and standard deviations).

	**Neutral**		**Toe-in**		**Toe-out**		**Lateral insoles**		**Conditions**
	**Mean**	*SD*	**Mean**	*SD*	**Mean**	*SD*	**Mean**	*SD*	
Extensor digitorum longus (W/kg)	0.08	0.02	0.08	0.03	0.08	0.02	0.08	0.02	
Gluteus maximus (W/kg)	0.25	0.06	0.24	0.06	0.26	0.07	0.24	0.05	***E, F***
Gluteus medius (W/kg)	0.37	0.14	0.32	0.14	0.42	0.11	0.37	0.14	***A, B, C, D, F***
Gluteus minimus (W/kg)	0.05	0.02	0.04	0.02	0.06	0.02	0.05	0.02	***A, B, C, D, F***
Psoas major (W/kg)	0.01	0.01	0.01	0.01	0.01	0.01	0.01	0.01	
Illiacus (W/kg)	0.02	0.01	0.01	0.01	0.01	0.01	0.02	0.02	***B, D***
Rectus femoris (W/kg)	0.00	0.00	0.00	0.00	0.01	0.01	0.01	0.01	
Vastus intermedius (W/kg)	0.06	0.01	0.05	0.01	0.05	0.01	0.06	0.01	***A, B, F***
Vastus lateralis (W/kg)	0.09	0.02	0.09	0.02	0.08	0.01	0.09	0.02	***A, B, F***
Vastus medialis (W/kg)	0.06	0.01	0.05	0.01	0.05	0.01	0.05	0.01	***A, B, F***
Adductor magnus (W/kg)	0.09	0.04	0.11	0.05	0.07	0.02	0.10	0.05	***A, B, C, E, F***
Biceps femoris long head (W/kg)	0.12	0.03	0.12	0.03	0.12	0.04	0.14	0.04	***B, C, D***
Biceps femoris short head (W/kg)	0.16	0.04	0.15	0.05	0.19	0.06	0.17	0.03	***A, C, D, F***
Semitendinosus (W/kg)	0.09	0.03	0.08	0.03	0.10	0.05	0.11	0.03	***C, D***
Semimembranosus (W/kg)	0.27	0.14	0.33	0.19	0.23	0.09	0.30	0.17	***B, C, E, F***
Sartorius (W/kg)	0.02	0.00	0.04	0.06	0.02	0.01	0.02	0.02	
Adductor longus (W/kg)	0.01	0.00	0.01	0.00	0.01	0.00	0.01	0.00	***A, E, F***
Tibialis anterior (W/kg)	0.18	0.04	0.17	0.07	0.17	0.05	0.17	0.05	
Medial gastrocnemius (W/kg)	0.17	0.04	0.17	0.05	0.19	0.05	0.17	0.03	***A, B, F***
Lateral gastrocnemius (W/kg)	0.04	0.01	0.04	0.01	0.05	0.01	0.05	0.01	
Soleus (W/kg)	0.19	0.04	0.19	0.05	0.18	0.04	0.16	0.04	***B, C, D***

A = Neutral significantly different from toe-out, B= Lateral insoles significantly different from toe-out, C = Lateral insoles significantly different from toe-in, D = Neutral significantly different from lateral insoles, E=Neutral significantly different from toe-in, F=Toe-in significantly different from toe-out.
